# Comprehensive Advances in General Medicine and Trauma Emergency Care: Integrating Surgery, Internal Medicine, and Lifestyle Medicine for Holistic Patient Management

**DOI:** 10.7759/cureus.114510

**Published:** 2026-08-13

**Authors:** Nametha Singh Jayasingh, Saba Aadil Memon, Shruthi K S, Geo George Thomas, Parv Rasiklal Raiyani, Himanshu Jha, Srinivas Babu Kathi

**Affiliations:** 1 Department of Medical Education, Dr. Lifeboat Medical Education, Thiruvananthapuram, IND; 2 Department of Mental Health and Addictions, St. Martha's Regional Hospital, Antigonish, CAN; 3 Department of Medicine, Rajiv Gandhi University of Health Sciences, Bengaluru, IND; 4 Department of Emergency Medicine, Christian Medical College, Vellore, Vellore, IND; 5 Department of Critical Care Medicine, Mahatma Gandhi Mission (MGM) New Bombay Hospital, Navi Mumbai, IND; 6 Department of Family Medicine, Down Town Hospital, Guwahati, IND; 7 Department of Pharmacy, Kaloji Narayana Rao University of Health Sciences, Warangal, IND

**Keywords:** critical care, emergency medicine, internal medicine, lifestyle medicine, trauma surgery

## Abstract

Emergency and trauma care increasingly involves patients with overlapping acute pathology, chronic disease, frailty, infection risk, functional limitation, and modifiable cardiometabolic factors. Although research published between 2020 and 2026 shows important advances in resuscitation, surgery, critical care, rehabilitation, and chronic disease prevention, these domains are often evaluated separately, limiting their translation into coordinated patient-centred care. This narrative review aimed to synthesise recent evidence from 2020 to 2026 on general medicine and trauma emergency care through an integrated framework combining surgery, internal medicine, rehabilitation, and lifestyle medicine. Studies were reviewed across trauma triage, prehospital and emergency resuscitation, operative and non-operative surgical decision-making, perioperative medical optimisation, sepsis management, organ support, analgesia, rehabilitation, lifestyle intervention, and digital follow-up. The evidence suggests that outcomes depend on accurate triage, timely stabilisation, appropriate use of resuscitative adjuncts, individualised surgical decisions, frailty assessment, comorbidity control, antimicrobial stewardship, physiologically guided critical care, multimodal analgesia, graded rehabilitation, and secondary prevention targeting nutrition, physical activity, glycaemic control, and cardiovascular risk. The literature also highlights uncertainties, including variable benefit from prehospital tranexamic acid and blood products, mixed findings on fluid restriction and sedation strategies, implementation barriers, and the need for longer-term functional outcome measures. Integrating emergency medicine, surgery, internal medicine, critical care, geriatrics, rehabilitation, digital health, and lifestyle medicine may improve survival, reduce complications, preserve function, and strengthen continuity of care.

## Introduction and background

General medicine and trauma emergency care have increasingly become integrated within a continuum extending from acute stabilisation and surgical decision-making to internal medicine optimisation, rehabilitation, and prevention. This review examines that continuum as a coordinated, patient-centred model linking emergency medicine, trauma and emergency surgery, internal medicine, critical care, rehabilitation, geriatrics, digital health, and lifestyle medicine. Lifestyle medicine refers to the structured use of evidence-based interventions involving nutrition, physical activity, weight management, tobacco and alcohol reduction, sleep, stress management, and other modifiable health behaviours to support recovery and reduce recurrent disease. Patients presenting to the emergency department may follow clinical trajectories that extend beyond the initial episode of care, including those with polytrauma, sepsis, myocardial infarction, stroke, metabolic decompensation, respiratory failure, abdominal emergencies, and injuries related to frailty and falls. Diseases and injuries continue to be major causes of death, disability-adjusted life years (DALYs), and years lived with disability (YLDs), according to the Global Burden of Disease (GBD) 2019 analysis, underscoring the need for systems that address both immediate threats and long-term disability [1]. Metabolic, behavioural, environmental, and occupational risk factors also influence patient vulnerability, illness or injury severity, treatment response, complications, and recovery following emergency and surgical care [2]. The objective of this review is to evaluate how acute stabilisation, surgical and medical management, rehabilitation, lifestyle intervention, and digitally supported follow-up can be coordinated to improve survival, reduce complications, preserve function, and strengthen continuity of care.

Chronic disease, acute illness, and injury frequently overlap in the emergency setting. Sepsis exemplifies this interaction because early recognition, antimicrobial therapy, haemodynamic resuscitation, source control, organ support, and critical care coordination all contribute to clinical outcomes [3]. Acute coronary syndromes, stroke, arrhythmias, heart failure, hypertensive crises, and perioperative cardiovascular complications are also major causes of emergency department presentation [4]. Communicable diseases, non-communicable diseases, and injuries collectively contribute to global morbidity and mortality, highlighting the need for systems capable of addressing overlapping clinical priorities [5].

Internal medicine plays a pivotal role in trauma and emergency surgical care because comorbidities influence risk stratification, operative tolerance, complications, and recovery. Diabetes is particularly relevant because its global prevalence is increasing and is projected to rise further [6]. Diabetes may increase infection risk, impair wound healing, exacerbate perioperative hyperglycaemia, destabilise cardiovascular status, compromise renal function, and prolong recovery after trauma or emergency surgery. In patients with trauma, intra-abdominal sepsis, postoperative infections, bloodstream infections, and ventilator-associated pneumonia, inappropriate or delayed antimicrobial treatment can worsen the clinical course, while antimicrobial resistance further complicates management [7]. Clinical assessment should therefore include the anatomical injury or surgical pathology, physiological status, immune function, renal function, medication exposure, nutritional reserve, and functional capacity.

Acute presentation and prognosis are also influenced by non-communicable diseases. The NCD Countdown 2030 analysis indicated that many countries were not on track to achieve reductions in premature mortality from major non-communicable diseases, reinforcing the need for stronger prevention strategies [8]. International Diabetes Federation estimates demonstrate the scale of the metabolic disease burden faced by emergency physicians, internists, surgeons, and intensivists [9]. Rehabilitation and functional recovery are also integral to care because musculoskeletal disorders such as osteoarthritis contribute to pain, immobility, falls, analgesic exposure, frailty, and postoperative functional limitation [10].

Long-term outcomes after emergency and trauma care are closely linked to cardiovascular risk reduction. Acute vascular events are associated with hypertension, dyslipidaemia, obesity, diabetes, smoking, unhealthy diet, and physical inactivity. These factors may also worsen perioperative and post-trauma recovery, particularly when presentation is delayed or resources are limited [11]. Country-level GBD data confirm the substantial cardiovascular morbidity and mortality attributable to modifiable risk factors, highlighting the need to extend care beyond stabilisation [12]. The American Heart Association's 2023 statistical update documents the substantial epidemiological burden of cardiovascular disease and stroke, including their contributions to mortality, morbidity, disability, and healthcare utilisation [13].

Survival alone is insufficient; comprehensive care must also prioritise prevention, functional recovery, and long-term health outcomes. Falls are a major cause of death, disability, and health loss worldwide, with a particularly important burden among older adults [14]. In clinical practice, fall risk may also be influenced by factors such as frailty, impaired mobility, multimorbidity, medication exposure, visual impairment, and environmental hazards. Occupational exposures also contribute to preventable injury and disease, making workplace prevention, exposure reduction, ergonomic measures, and policy interventions essential [15]. Chronic disease patterns, environmental exposures, and social determinants of health are therefore reflected in many emergency and trauma presentations.

Although advances in trauma systems, emergency resuscitation, critical care, surgery, and chronic disease management have improved survival, the supporting evidence is commonly evaluated within separate disciplinary domains. Few contemporary reviews have synthesised how prehospital care, acute stabilisation, operative and non-operative decision-making, internal medicine co-management, critical care, rehabilitation, lifestyle-based secondary prevention, geriatric assessment, and digital follow-up should be coordinated across the complete continuum of emergency and trauma care. This represents an important research gap because fragmented evidence may support individual interventions without clarifying how they should be sequenced or integrated in practice. Fragmented care may address the immediate crisis while giving insufficient attention to comorbidities, functional limitations, behavioural risks, and environmental drivers.

Lifestyle medicine should be introduced according to the patient's clinical phase. During the immediate post-acute period, appropriate measures include nutritional assessment and optimisation, gradual mobilisation within physiological limits, glycaemic management, medication-adherence planning, and brief counselling regarding tobacco and harmful alcohol use. More intensive interventions, including structured dietary modification, progressive physical activity, weight management, sustained tobacco and alcohol reduction, sleep optimisation, stress management, and long-term chronic disease prevention, are more appropriately implemented during rehabilitation and outpatient follow-up.

**Figure 1:**
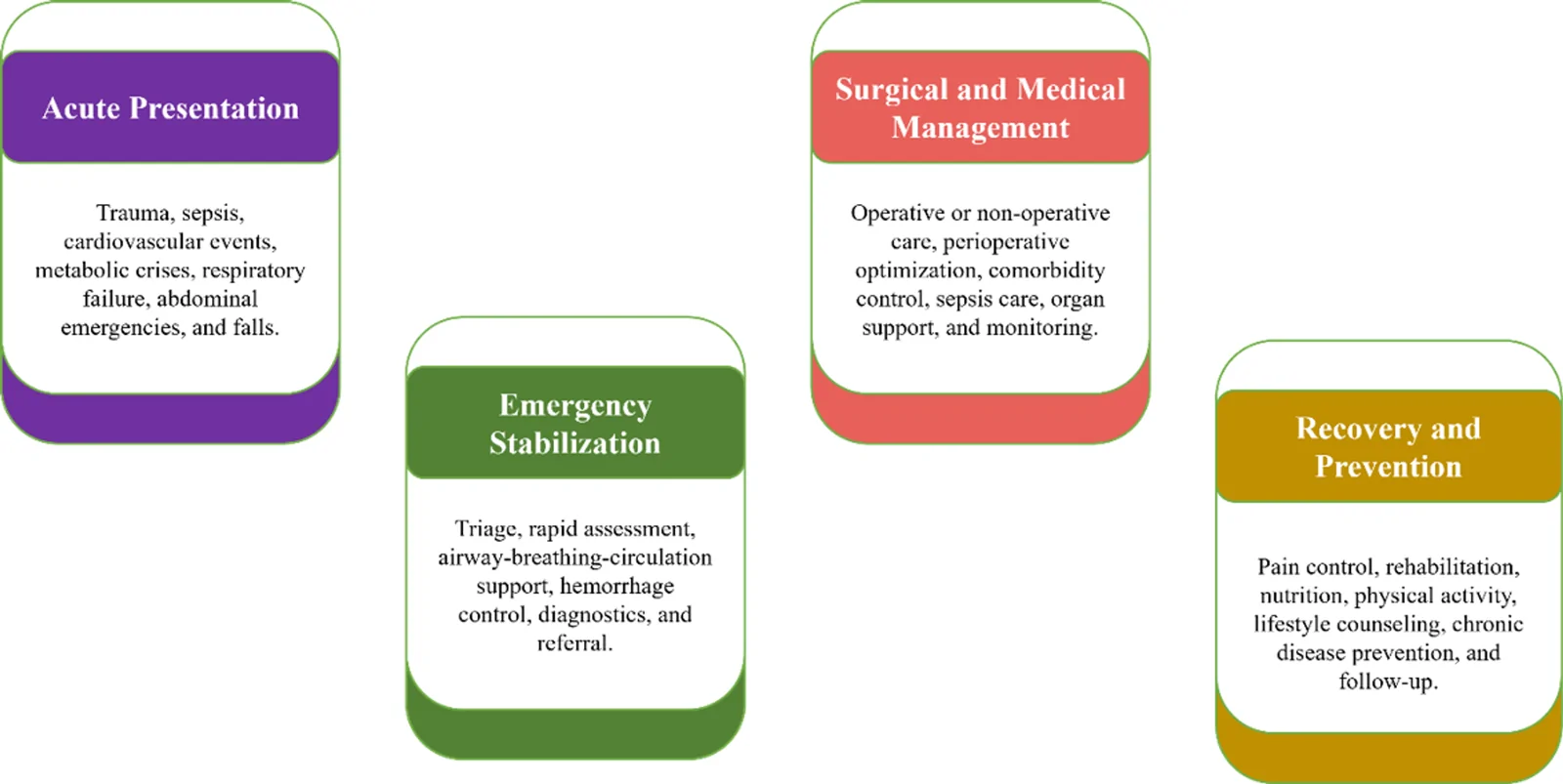
Integrated continuum of holistic emergency and trauma care The framework illustrates the progression from acute stabilisation and medical-surgical management to rehabilitation, chronic disease control, risk-factor modification, and long-term prevention [1-4,8-15]. The image was created by the authors using Microsoft PowerPoint (Microsoft Corporation, Redmond, Washington, United States) based on the cited literature.

A narrative review was selected because the available evidence encompasses heterogeneous populations, interventions, clinical settings, study designs, and outcomes that are not suitable for a single pooled quantitative analysis. This approach permits an integrated interpretation of evidence across emergency medicine, surgery, internal medicine, rehabilitation, and lifestyle medicine. The review was therefore undertaken to address the identified gap by synthesising recent evidence from 2020 to 2026 within a unified framework spanning acute care, recovery, chronic disease control, secondary prevention, and long-term patient-centred outcomes. The proposed continuum of care is illustrated in Figure 1.

Objectives of the review

This review examines recent advances in general medicine and trauma emergency care through an integrated, multidisciplinary, and patient-centred framework. It evaluates evidence across prehospital triage, early resuscitation, operative and non-operative surgical decision-making, internal medicine co-management, critical care, infection control, analgesia, rehabilitation, perioperative optimisation, lifestyle-based secondary prevention, geriatric care, and digital follow-up. The review further assesses how these components can be coordinated across the continuum of care to improve survival and recovery, reduce complications, preserve function, strengthen chronic disease control, and prevent recurrent emergency presentations.

Methodology

Literature Search and Review Design

A structured narrative literature search was undertaken to identify contemporary evidence relevant to the integration of general medicine, trauma emergency care, surgery, critical care, rehabilitation, and lifestyle medicine. A narrative synthesis was selected because the review encompassed clinically and methodologically heterogeneous populations, interventions, settings, study designs, and outcome measures that were unsuitable for a single pooled quantitative analysis.

Search Strategy and Information Sources

PubMed/MEDLINE, Scopus, Web of Science, and Google Scholar were searched for articles published from January 1, 2020, to July 31, 2026. The search period was selected to capture recent developments in trauma systems, resuscitation, emergency surgery, perioperative medicine, sepsis management, organ support, analgesia, rehabilitation, chronic disease control, and digitally supported follow-up. Reference lists of eligible articles were also reviewed to identify additional relevant publications.

Search terms were combined using the Boolean operators "AND" and "OR". Principal concepts included "trauma", "emergency care", "emergency medicine", "trauma surgery", "emergency general surgery", "prehospital care", "triage", "resuscitation", "haemorrhagic shock", "tranexamic acid", "blood products", "sepsis", "critical care", "organ support", "frailty", "perioperative medicine", "internal medicine co-management", "pain management", "regional analgesia", "rehabilitation", "early mobilisation", "lifestyle medicine", "secondary prevention", "Mediterranean diet", "diabetes remission", "prehabilitation", "geriatric trauma", "digital health", and "postoperative monitoring".

Searches were adapted to the indexing structure and syntax of each database. An illustrative search strategy was: (“trauma” OR “emergency care” OR “emergency surgery”) AND (“resuscitation” OR “critical care” OR “internal medicine” OR “rehabilitation” OR “lifestyle medicine” OR “digital follow-up”). Additional focused searches were performed for individual interventions and clinical domains. Because complete database-specific search histories were not retained, the reported strategy represents the principal search framework rather than a fully reproducible record of every query conducted.

Eligibility Criteria

Studies were eligible when they were published in English; involved patients receiving emergency, trauma, surgical, perioperative, critical care, rehabilitation, or post-discharge care; reported clinically relevant outcomes such as mortality, complications, functional recovery, delirium, mobilisation, pain, recurrence, quality of life, healthcare utilisation, or chronic disease control; evaluated an intervention, care model, prognostic factor, health-system characteristic, or preventive strategy relevant to the review objectives; and used a randomised controlled, prospective cohort, retrospective cohort, systematic review, meta-analysis, large epidemiological, or other clinically substantive design.

Citation selection prioritised multicentre investigations, randomised controlled trials, systematic reviews, meta-analyses, international clinical guidelines, and large population-based studies. Where several publications addressed the same clinical question, preference was given to the most recent, methodologically robust, and directly relevant source.

Articles were excluded when they were unrelated to emergency, trauma, surgical, medical, rehabilitative, or preventive care; involved exclusively laboratory, animal, or preclinical research; were conference abstracts without adequate methodological or outcome data; were case reports, opinion pieces, letters, or narrative commentaries without substantive clinical evidence; duplicated data reported in another included publication; or did not contribute directly to the integrated patient-centred framework of the review.

Publications before 2020 were generally excluded. One earlier randomised clinical trial on mobile postoperative monitoring was retained because it provided directly relevant evidence for digitally supported follow-up and because no comparable recent study addressing the same specific application was identified.

Study Selection

Titles and abstracts were assessed for relevance to the review objectives, followed by full-text evaluation of potentially eligible publications. Studies were selected according to clinical relevance, methodological quality, recency, and contribution to one or more prespecified domains: trauma-system organisation and triage, initial assessment and resuscitation, surgical decision-making, internal medicine and frailty assessment, sepsis and critical care, pain management, rehabilitation, lifestyle-based secondary prevention, geriatric care, and digital monitoring.

The selection process was intended to provide representative, high-quality evidence across each domain rather than exhaustive coverage of every publication.

Data Extraction

For each included source, information was extracted on the author, publication year, study design, clinical population, care setting, intervention or exposure, comparator where applicable, principal outcomes, reported effect estimates, follow-up period, and key methodological limitations.

Extracted evidence was assigned to the prespecified clinical domains and organised according to the phase of care, intervention type, and relevance to the integrated emergency and trauma framework.

Narrative Synthesis

Narrative synthesis was conducted by comparing studies within each domain according to population characteristics, study design, intervention, comparator, outcome definition, follow-up period, direction of effect, and clinical applicability.

Randomised controlled trials, systematic reviews, meta-analyses, international guidelines, and large multicentre investigations were given greater interpretive weight where available. Observational studies were used primarily to describe prognostic associations, implementation considerations, and clinical areas not adequately addressed by randomised evidence.

Findings were classified as beneficial, neutral, mixed, or potentially harmful. Apparent inconsistencies were interpreted in relation to differences in patient selection, intervention timing, treatment intensity, comparator groups, outcome definitions, and duration of follow-up. No quantitative meta-analysis was undertaken because the included evidence was clinically and methodologically heterogeneous.

Methodological Appraisal

Because this was a narrative review, formal risk-of-bias scoring and certainty-of-evidence grading were not performed. Methodological quality was instead appraised qualitatively according to study design, sample size, multicentre participation, relevance of outcomes, consistency of findings, and direct applicability to the review objectives.

Scale for the Assessment of Narrative Review Articles (SANRA) Assessment

The final manuscript was assessed using SANRA [15]. The assessment considered six domains: justification of the review's importance, statement of concrete aims, description of the literature search, referencing, scientific reasoning, and presentation of relevant endpoint data. The completed SANRA checklist was provided in the Appendices.

Included Evidence and Replicability Limitation

The final synthesis included 47 sources comprising global burden and epidemiological analyses, randomised controlled trials, cohort studies, systematic reviews, meta-analyses, and international clinical guidance. Evidence was organised thematically and interpreted according to its implications for integrated emergency and trauma care, with particular attention to survival, complications, functional recovery, continuity of care, and long-term prevention.

Complete database-specific retrieval counts, deduplication records, and screening logs were not retained. Therefore, exact replication of the original search and study-selection process was not possible and is acknowledged as a limitation.

## Review

Evolution of trauma and emergency care systems

From episodic, hospital-centred responses, trauma and emergency care systems have developed into coordinated systems that focus on early recognition, accurate triage, timely transport to an appropriate facility, and multidisciplinary response. Prehospital triage continues to be fundamental to system performance because transporting an injured patient to an appropriate trauma centre affects access to definitive imaging, surgery, blood products, and critical care. Lokerman et al. demonstrated that a structured trauma-triage intervention improved the identification of severely injured adults in the prehospital setting, supporting the use of standardised field assessment, decision-support tools, and feedback between prehospital services and trauma centres [16]. Improved triage accuracy alone may not improve clinical outcomes unless it is accompanied by adequate trauma-centre resources, infrastructure, timely handover, and access to definitive care.

Emergency-system performance is also increasingly evaluated across age groups and specialised care environments. Greater emergency department paediatric readiness was associated with reduced short- and long-term mortality among children receiving emergency care, underscoring the importance of appropriate protocols, trained staff, equipment, and age-specific resources [17]. These findings suggest that emergency preparedness should not be limited to designated trauma centres and that minimum readiness standards are also needed in community and non-specialist hospitals. Implementation remains challenging in resource-limited settings because shortages of staff and equipment, together with inconsistent referral pathways, may reduce the effectiveness of guideline-based readiness initiatives.

The development of trauma and emergency care systems is also influenced by the institutions responsible for research priority-setting, guideline development, professional education, and dissemination of surgical evidence. Evidence of gender underrepresentation on international general surgery editorial boards highlights the need for greater diversity within these structures to support inclusive research agendas and greater attention to population-specific clinical needs [18].

Quality assessment in trauma care has also expanded beyond immediate survival. This review's synthesis is consistent with the findings of Zogg et al., who showed that assessments of trauma-care performance changed when outcomes among older adults were evaluated using longer-term measures rather than in-hospital mortality alone [19]. This is especially relevant because older adults with trauma may survive hospitalisation but subsequently experience disability, institutionalisation, readmission, or loss of independent living. Modern trauma systems should therefore be evaluated not only by survival and timeliness of care but also by equity, readiness, patient-centred functional recovery, and long-term outcomes.

Initial assessment, resuscitation, and stabilisation of the acutely ill patient

Identifying and managing potentially reversible threats to oxygenation, ventilation, circulation, perfusion, and neurological function are critical components of initial trauma resuscitation. This structured, priority-based approach is consistent with Advanced Trauma Life Support principles, which emphasise systematic primary assessment, immediate treatment of life-threatening abnormalities, repeated reassessment, and timely transfer to definitive care [20]. Contemporary trauma care has increasingly evaluated out-of-hospital interventions, particularly in patients with suspected haemorrhage or traumatic brain injury. Rowell et al. assessed out-of-hospital tranexamic acid in patients with moderate-to-severe traumatic brain injury and found no significant improvement in six-month functional neurological outcomes compared with placebo (65% versus 62%; absolute difference: 3.5%; 90% CI: -0.9% to 7.9%; p=0.16) [21]. This finding does not support the indiscriminate use of antifibrinolytic therapy in traumatic brain injury and indicates that patient selection, injury phenotype, treatment timing, and clinically meaningful outcome measures should guide interpretation and clinical application.

In patients at risk of traumatic haemorrhage, Guyette et al. evaluated tranexamic acid administered during prehospital transport and found no significant reduction in 30-day mortality in the overall cohort (8.1% with tranexamic acid versus 9.9% with placebo; difference: -1.8%; 95% CI: -5.6% to 1.9%; p=0.17), although selected subgroup analyses suggested possible benefit [22]. The PATCH-Trauma trial found that prehospital tranexamic acid did not improve survival with a favourable functional outcome at six months (53.7% versus 53.5%; risk ratio: 1.00; 95% CI: 0.90-1.12; p=0.95), although 28-day mortality was lower in the tranexamic acid group (17.3% versus 21.8%; risk ratio: 0.79; 95% CI: 0.63-0.99) [23]. These findings should be interpreted within a broader trauma-resuscitation pathway in which pharmacological adjuncts complement, rather than replace, rapid haemorrhage identification, mechanical or surgical bleeding control, balanced resuscitation, and timely definitive intervention. Tranexamic acid should therefore be considered a targeted adjunct for appropriately selected patients rather than a substitute for definitive haemorrhage control.

Haemorrhagic-shock resuscitation has increasingly focused on the early restoration of oxygen-carrying capacity and correction of trauma-associated coagulopathy. In the RePHILL trial, prehospital packed red blood cells and lyophilised plasma did not improve the composite outcome of mortality or failure of lactate clearance compared with standard care (64% versus 65%; adjusted risk ratio: 1.01; 95% CI: 0.88-1.17) [24]. This neutral finding does not negate the role of blood-component therapy in definitive trauma care but highlights the logistical, timing, and patient-selection challenges associated with prehospital blood-product administration. Initial trauma management should therefore integrate structured assessment, rapid haemorrhage control, physiological monitoring, selective use of resuscitative adjuncts, repeated reassessment, and timely transfer to an appropriately resourced trauma centre. Resuscitation evidence is summarised in Table 1.

**Table 1:** Key evidence on early trauma stabilisation

Theme	Key content	Critical interpretation	References
Initial assessment and stabilisation	Advanced Trauma Life Support principles support systematic primary assessment, immediate treatment of life-threatening abnormalities, repeated reassessment, and timely transfer to definitive care.	Early stabilisation should remain structured and physiology-driven, with prehospital interventions used only when they facilitate rather than delay definitive care.	[20]
Tranexamic acid in traumatic brain injury	Out-of-hospital tranexamic acid did not significantly improve six-month functional neurological outcomes in moderate-to-severe traumatic brain injury.	Routine antifibrinolytic treatment is not supported for all patients with traumatic brain injury; selection should consider injury phenotype, timing, and clinically meaningful outcomes.	[21]
Tranexamic acid in haemorrhage-risk trauma	Prehospital tranexamic acid did not significantly reduce 30-day mortality in the overall haemorrhage-risk trauma population.	Tranexamic acid should be considered a selective adjunct and should not replace rapid haemorrhage control, blood-product resuscitation, or definitive intervention.	[22]
PATCH-Trauma trial	Prehospital tranexamic acid reduced 28-day mortality but did not improve survival with a favourable functional outcome at six months.	Early survival benefit may not translate into sustained functional recovery; long-term patient-centred outcomes remain essential.	[23]
Prehospital blood-product resuscitation	In the RePHILL trial, prehospital packed red blood cells and lyophilised plasma did not improve the composite outcome of mortality or failure of lactate clearance.	The findings highlight logistical, timing, and patient-selection challenges rather than negating the role of balanced transfusion in definitive trauma care.	[24]
Overall stabilisation approach	Effective stabilisation integrates structured assessment, haemorrhage control, physiological monitoring, selective adjuncts, reassessment, and timely transfer.	Resuscitation requires coordination across prehospital care, emergency assessment, definitive haemorrhage control, and critical care transition.	[20-24]

Surgical decision-making in trauma and emergency general surgery

Decision-making in trauma and emergency general surgery is no longer based on an automatic operative response to every injury or acute surgical condition. Instead, management is increasingly selected according to the patient's physiological status, disease characteristics, treatment risks, and individual preferences. The CODA trial found that antibiotics were non-inferior to appendectomy with respect to 30-day health status (mean EQ-5D difference: 0.01 points; 95% CI: -0.001 to 0.03). Within 90 days, 29% of patients assigned to antibiotics underwent appendectomy, including 41% of those with an appendicolith and 25% of those without an appendicolith [25]. These findings support shared decision-making in appropriately selected patients while emphasising the need to discuss recurrence, treatment failure, and the potential requirement for later surgery.

The APPAC II trial compared oral moxifloxacin with intravenous ertapenem followed by oral levofloxacin and metronidazole for uncomplicated acute appendicitis [26]. Although oral therapy did not meet the prespecified criterion for non-inferiority, one-year treatment success occurred in 70.2% of the oral-treatment group and 73.8% of the intravenous-followed-by-oral group (difference: -3.6%; one-sided 95% CI: -9.7% to infinity; p=0.26). The trial remains clinically relevant because it evaluated whether an entirely oral outpatient regimen could replace treatment requiring initial intravenous administration. The findings illustrate the broader objective of reducing treatment burden without compromising efficacy, recurrence risk, antimicrobial stewardship, or patient safety. They do not, however, establish oral monotherapy as equivalent to the combined intravenous and oral regimen.

Traumatic injuries may also result in severe physiological compromise requiring rapid haemorrhage-control decisions. In the UK-REBOA trial, investigators evaluated resuscitative endovascular balloon occlusion of the aorta for exsanguinating haemorrhage and found that 90-day mortality was 54% with REBOA plus standard care and 42% with standard care alone (odds ratio: 1.58; posterior probability of increased mortality with REBOA: 86.9%) [27]. This neutral and potentially harmful finding does not support routine use of REBOA and reinforces the need for careful patient selection, appropriate expertise, and timely definitive haemorrhage control. Patient-related factors are also central to surgical decision-making. Houghton et al. found that frailty was associated with worse outcomes following vascular surgery, indicating that outcomes in acutely ill surgical patients are determined not only by the underlying pathology but also by physiological reserve, multimorbidity, and functional vulnerability [28].

Internal medicine integration in acute surgical and trauma patients

Internal medicine integration is essential in acute surgical and trauma care because patient outcomes depend not only on the injury or operative pathology but also on physiological status, multimorbidity, frailty, medication exposure, and organ reserve. McIsaac et al. described frailty as a multidimensional syndrome that is important for surgeons and anaesthetists to assess preoperatively because it is associated with greater vulnerability to complications, delayed recovery, institutional discharge, and mortality [29]. Frailty assessment should therefore inform risk communication, shared decision-making, perioperative optimisation, postoperative monitoring, rehabilitation planning, and individualised care.

Evidence from high-acuity surgical populations further supports coordinated medical and surgical management. The HIP ATTACK trial, which randomised 2,970 patients, found that accelerated surgery for hip fracture did not reduce mortality or the composite outcome of death and major complications compared with standard care [30]. However, accelerated surgery was associated with less delirium and earlier mobilisation and readiness for discharge. These findings suggest that earlier surgery may improve selected recovery-related outcomes but should not be interpreted as sufficient on its own. Its benefits depend on concurrent medical stabilisation, effective analgesia, delirium prevention, fluid and haemodynamic management, thromboprophylaxis, and timely rehabilitation.

Frailty should be interpreted as a marker of reduced physiological reserve rather than as a condition confined to older surgical patients. In the COPE registry, frailty was associated with lower survival among hospitalised patients with coronavirus pneumonia in a multicentre cohort of 1,564 patients [31]. This finding demonstrates that frailty is relevant across acute medical and surgical illness, including patients who do not undergo an operation. Castillo-Angeles et al. examined 882,929 emergency general surgery admissions and found that frailty was associated with increased morbidity and mortality across procedural-risk categories; among low-risk procedures, frailty was associated with approximately twice the adjusted odds of mortality (odds ratio: 2.05; 95% CI: 1.94-2.17) [32].

These findings support a focused role for internal medicine co-management in identifying physiological vulnerability, optimising chronic disease and medication exposure, assessing cardiorespiratory and renal reserve, preventing delirium and thromboembolic complications, and coordinating postoperative recovery. Comprehensive geriatric consultation, including cognitive assessment, medication review, discharge safety, and post-acute service coordination, is addressed separately in the integrated care section to avoid duplicating the discussion of frailty.

Critical care, sepsis, organ support, and postoperative monitoring

Critically ill patients transition from emergency stabilisation to recovery through organ support, prevention of secondary injury, and early recognition of complications associated with trauma, critical illness, and emergency surgery. Current Surviving Sepsis Campaign guidance supports early recognition and treatment of sepsis, timely antimicrobial therapy, source control, initial crystalloid resuscitation when hypoperfusion is present, repeated assessment of fluid responsiveness, and vasopressor support when hypotension persists [33]. These recommendations support an individualised, physiology-guided approach rather than uniform fluid administration for every patient.

Fluid management is central to septic shock care but remains clinically complex. Meyhoff et al. found that restrictive intravenous fluid therapy, compared with standard fluid therapy, did not significantly reduce 90-day mortality among intensive care unit patients with septic shock (42.3% versus 42.1%; adjusted absolute difference: 0.1 percentage points; 95% CI: -4.7 to 4.9) [34]. This finding does not support rigid, one-size-fits-all fluid-restriction protocols and instead highlights the importance of individualising fluid therapy according to perfusion status, vasopressor requirements, cardiorenal function, cumulative fluid exposure, and haemodynamic response.

The CLOVERS trial similarly found no significant difference in mortality before discharge home by day 90 between restrictive and liberal fluid strategies among patients with sepsis-induced hypotension (14% versus 14.9%; estimated difference: -0.9 percentage points; 95% CI: -4.4 to 2.6) [35]. These neutral findings do not eliminate the need for early resuscitation but indicate that neither restrictive nor liberal fluid administration should be applied indiscriminately. Fluid decisions should be guided by repeated clinical and haemodynamic assessment, with prompt vasopressor initiation when adequate perfusion cannot be maintained through appropriate fluid resuscitation alone. Sepsis management should also integrate antimicrobial therapy, source control, perfusion monitoring, organ support, and prevention of both fluid overload and inadequate resuscitation.

Sedation is another important component of critical care. Hughes et al. compared propofol with dexmedetomidine in mechanically ventilated adults with sepsis and found no significant difference in days alive without delirium or coma (adjusted median: 10.8 versus 10.7 days; odds ratio: 0.96; 95% CI: 0.74-1.26) [36]. This finding indicates that sedative selection alone is unlikely to prevent delirium, which is influenced by sepsis, hypoxia, pain, sleep disruption, immobility, organ dysfunction, medication exposure, and other patient-specific factors. Sedation should therefore be individualised and incorporated into a broader strategy involving regular assessment, avoidance of unnecessary deep sedation, pain control, delirium prevention, and early liberation from mechanical ventilation when clinically feasible.

**Figure 2:**
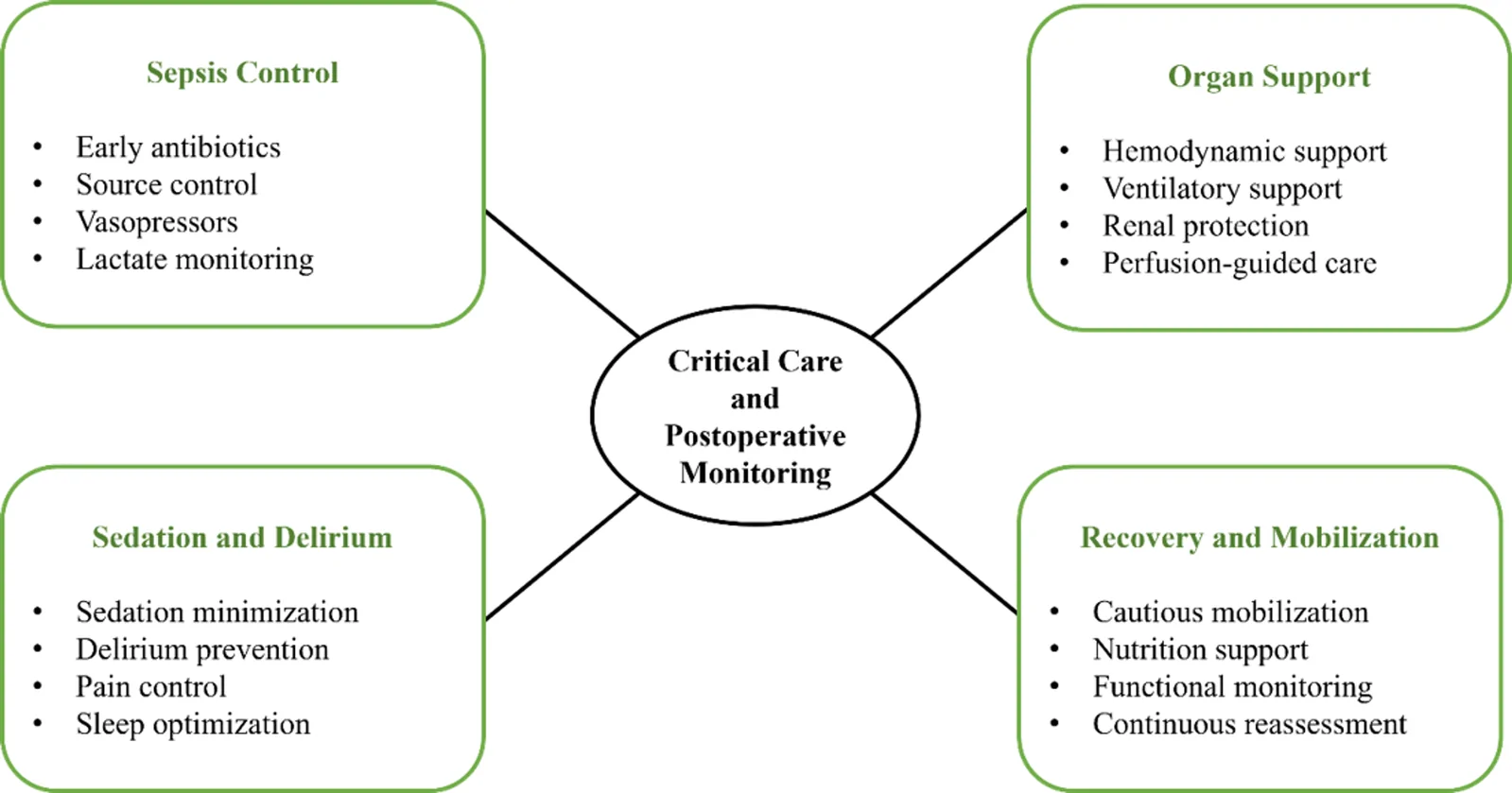
Core components of critical care, sepsis management, organ support, and postoperative monitoring The framework summarises sepsis control, haemodynamic and organ support, sedation and delirium management, and cautious recovery and mobilisation [32-35]. The image was created by the authors using Microsoft PowerPoint (Microsoft Corporation, Redmond, Washington, United States) based on the cited literature.

The timing and intensity of rehabilitation also require careful consideration. The TEAM trial found that early active mobilisation during mechanical ventilation did not improve days alive and out of hospital compared with usual mobilisation, while mobilisation-related adverse events occurred more frequently (9.2% versus 4.1%; p=0.005) [37]. This finding does not argue against rehabilitation but cautions against intensive mobilisation before adequate physiological stability has been achieved. Mobilisation should be introduced gradually according to haemodynamic stability, respiratory support, neurological status, functional reserve, and tolerance. Management of severe illness should therefore combine timely sepsis control, organ protection, individualised haemodynamic support, appropriate sedation and analgesia, delirium-risk reduction, adequate nutrition, cautious mobilisation, and frequent reassessment. Figure 2 presents this integrated critical care framework.

Pain management, rehabilitation, and functional recovery

Pain relief is an essential component of trauma and emergency surgical care because untreated pain can impair respiratory function, restrict mobilisation, activate physiological stress responses, disrupt sleep, and contribute to delirium or chronic pain. Tekşen et al. found that the serratus anterior plane block provided effective analgesia in patients with rib fractures [38]. Regional analgesia may be particularly useful in thoracic trauma because improved pain control can facilitate coughing, deep breathing, mobilisation, and participation in physiotherapy. Its effectiveness, however, depends on appropriate patient selection, clinician expertise, ultrasound availability, and adequate monitoring.

Chen et al. found that flurbiprofen axetil combined with ultrasound-guided intercostal nerve block improved preoperative analgesia in patients with traumatic multiple rib fractures [39]. These findings suggest that multimodal analgesia combining regional anaesthesia with non-opioid medication may improve pain control and reduce reliance on systemic opioids. Anti-inflammatory agents are not suitable for every patient, particularly those with renal impairment, gastrointestinal disease, increased bleeding risk, or other perioperative contraindications.

Evidence also supports opioid-sparing strategies to reduce complications associated with opioid exposure. Zhang et al. reported that opioid-sparing analgesia improved postoperative pain and recovery outcomes across randomised controlled trials [40]. Opioid reduction may decrease nausea, ileus, sedation, respiratory depression, dependence risk, and delayed mobilisation. Opioid-sparing care should not be interpreted as complete opioid avoidance, and analgesic regimens should be individualised according to injury severity, surgical complexity, comorbidities, contraindications, and functional goals.

Rehabilitation is central to functional recovery but requires adequate pain control and physiological stability. Glinsky et al. evaluated intensive task-specific training after recent spinal cord injury, highlighting the importance of training intensity, task relevance, patient selection, and safety monitoring [41]. Rehabilitation should therefore extend beyond analgesia to include graded mobilisation, respiratory exercises, nutritional support, psychological care, functional goal-setting, and discharge planning. The principal components of pain management and functional recovery are summarised in Table 2.

**Table 2:** Evidence summary of pain control and functional recovery

Theme	Key content	Critical interpretation	References
Pain control in trauma care	Effective analgesia supports respiratory function, mobilisation, sleep, rehabilitation participation, and recovery after trauma or emergency surgery.	Pain management should be treated as a functional intervention rather than as symptom control alone.	[38-41]
Serratus anterior plane block	The serratus anterior plane block provided effective analgesia in patients with rib fractures.	Regional analgesia may reduce systemic opioid exposure and facilitate coughing, deep breathing, mobilisation, and physiotherapy when appropriate expertise and monitoring are available.	[38]
Multimodal analgesia in rib fractures	Flurbiprofen axetil combined with ultrasound-guided intercostal nerve block improved preoperative analgesia in traumatic multiple rib fractures.	Regional techniques combined with non-opioid medication may improve pain control, but anti-inflammatory therapy requires consideration of renal, gastrointestinal, bleeding, and perioperative risks.	[39]
Opioid-sparing strategies	Opioid-sparing analgesia was associated with improved postoperative pain and recovery outcomes across randomised trials.	Opioid reduction may limit treatment-related adverse effects, but it should not result in complete opioid avoidance or undertreatment of pain.	[40]
Functional rehabilitation	Intensive task-specific training after recent spinal cord injury highlighted the importance of rehabilitation intensity, task relevance, patient selection, and safety.	Rehabilitation should incorporate graded mobilisation, respiratory exercises, nutritional support, psychological care, functional goals, and discharge planning.	[41]
Integrated recovery approach	Multimodal analgesia and structured rehabilitation should be introduced according to physiological stability and individual recovery needs.	The primary objective is restoration of function rather than reduction of pain scores alone.	[38-41]

Lifestyle medicine in recovery, secondary prevention, and chronic disease control

After clinical stabilisation, the focus of care progressively shifts from immediate survival to functional recovery, recurrence prevention, and long-term disease control. In the randomised CORDIOPREV trial involving patients with coronary heart disease, a Mediterranean diet was associated with a lower incidence of major cardiovascular events than a low-fat diet during long-term secondary prevention [42]. This evidence is relevant to emergency and post-acute care because many hospital presentations, including myocardial infarction, stroke, and perioperative cardiovascular complications, are associated with modifiable cardiometabolic risk factors.

Dietary intervention should therefore form part of structured secondary prevention rather than being limited to general patient education. Lifestyle medicine can also contribute to metabolic recovery. The DIADEM-I trial demonstrated that intensive lifestyle intervention promoted weight loss and improved glycaemic control in patients with early type 2 diabetes [43]. These strategies may benefit patients whose recovery is adversely affected by obesity, hyperglycaemia, increased infection risk, and impaired wound healing. Their implementation remains challenging because sustained behavioural change requires continued motivation, structured support, and regular follow-up. Compared with calorie restriction plus placebo, dapagliflozin combined with calorie restriction resulted in a significantly higher rate of type 2 diabetes remission [44].

This finding suggests that pharmacological therapy may augment lifestyle-based interventions in appropriately selected patients. Medication-supported strategies should nevertheless be individualised according to treatment response, comorbidities, contraindications, and adverse-effect risk, with continued clinical monitoring. An energy-reduced Mediterranean diet combined with physical activity also produced changes in the gut metabolome and microbiota after one year [45]. These findings suggest that sustained lifestyle modification may produce biological effects beyond weight loss, including changes in metabolic and microbial profiles. Lifestyle medicine may therefore complement surgery, internal medicine, and emergency care through nutritional optimisation, physical activity, weight management, glycaemic control, cardiovascular risk reduction, and prevention of recurrent illness.

Integrated holistic model for patient-centred emergency and trauma care

Acute stabilisation, perioperative optimisation, rehabilitation, chronic disease management, digital monitoring, and preventive care are interconnected components of an integrated approach to emergency and trauma management. Available evidence suggests that prehabilitation may improve readiness for surgery through exercise, nutritional support, psychological interventions, and multimodal optimisation [46]. Although formal prehabilitation is difficult to implement in trauma and emergency surgery because of limited preparation time, its principles can be adapted after stabilisation through early nutritional assessment, graded functional conditioning, respiratory exercises, medication optimisation, and psychological support.

Patient-centred care also entails considering patients' access to and understanding of health information before hospital presentation. Self-triage applications may be useful for young adults; however, concerns about inaccuracy, anxiety, and over-reliance on digital tools were also reported by this age group [47]. Digital technologies should complement clinical assessment rather than replace it, particularly in patients presenting with trauma, sepsis, chest pain, neurological deficits, or respiratory distress. The complex needs of older adults should also be incorporated into trauma systems. Geriatric consultation has been associated with improved outcomes among older adults presenting with traumatic injuries [48].

Support for older adults should include comprehensive geriatric assessment, frailty screening, medication review, delirium and cognitive-risk assessment, rehabilitation planning, and coordinated discharge. After ambulatory surgery, postoperative monitoring through mobile applications has been shown to reduce the need for in-person follow-up visits [49]. Although publications from 2020 to 2026 were prioritised, one earlier randomised clinical trial was retained because it provided directly relevant evidence on mobile application-based postoperative monitoring and no comparable recent study addressing the same specific application was identified. This exception and its rationale are stated explicitly in the Methodology subsection. Digital follow-up should nevertheless be implemented selectively, with consideration of digital literacy, access to technology, clinical escalation pathways, and patient safety. Surgery, internal medicine, rehabilitation medicine, lifestyle medicine, geriatric medicine, and digital healthcare should therefore be integrated into holistic emergency and trauma care to improve survival, functional recovery, continuity of care, and long-term outcomes. The integrated components of patient-centred emergency and trauma care are summarised in Table 3.

**Table 3:** Integrated model for holistic emergency and trauma care

Theme	Key content	Critical interpretation	References
Integrated care model	Holistic emergency and trauma care links acute stabilisation with perioperative optimisation, rehabilitation, chronic disease control, digital follow-up, and prevention.	Care should extend beyond survival and discharge to address recovery readiness, recurrence prevention, function, and continuity of care.	[46-49]
Prehabilitation principles	Prehabilitation may improve surgical preparedness through exercise, nutritional support, psychological interventions, and multimodal optimisation.	Formal prehabilitation is often impractical in emergency surgery, but its principles can be adapted after stabilisation through nutritional assessment, graded conditioning, respiratory exercises, medication optimisation, and psychological support.	[46]
Digital self-triage	Young adults viewed symptom checkers as potentially useful but raised concerns about accuracy, anxiety, and over-reliance.	Digital tools may support access and preliminary decision-making but should not replace clinical assessment for high-risk presentations.	[47]
Geriatric trauma care	Geriatric consultation was associated with improved outcomes among older adults with traumatic injuries.	Comprehensive geriatric input should focus on medication review, cognition, delirium risk, rehabilitation planning, discharge safety, and care coordination rather than duplicating frailty assessment.	[48]
Mobile postoperative monitoring	Mobile application-based home monitoring reduced the need for in-person follow-up after ambulatory surgery.	Digital follow-up may improve surveillance and reduce unnecessary visits, but safe implementation depends on patient selection, digital literacy, escalation pathways, and access to technology.	[49]
Patient-centred outcome focus	Holistic care integrates surgery, internal medicine, rehabilitation, lifestyle medicine, geriatric input, and digital follow-up.	The aim is to improve survival, function, continuity, patient engagement, quality of life, and prevention of recurrent illness through individualised pathways.	[46-49]

Limitations and future directions

This narrative review has a few limitations. The broad scope required synthesis across heterogeneous populations, interventions, care settings, study designs, and outcome measures, which limited direct comparison and precluded quantitative pooling. The search was restricted to English-language publications and did not include a formal risk-of-bias or certainty-of-evidence assessment, which may have introduced selection bias and limited the strength of evidence appraisal. Evidence from randomised trials, observational studies, epidemiological analyses, systematic reviews, meta-analyses, and clinical guidance was combined within one framework, and some domains were represented by only a small number of studies. Complete numerical records of citations identified, deduplicated, screened, and excluded were not retained; therefore, a Preferred Reporting Items for Systematic Reviews and Meta-Analyses (PRISMA)-style study-selection flow could not be reported or reconstructed reliably. One pre-2020 trial was retained because of its specific relevance to mobile postoperative monitoring. The proposed integrated model should therefore be interpreted as a clinically informed synthesis rather than a definitive evidence-based pathway.

Future research should evaluate integrated emergency and trauma pathways prospectively across diverse healthcare systems. Priorities include longer-term functional and quality-of-life outcomes, cost-effectiveness, implementation in resource-limited settings, and identification of patients most likely to benefit from specific interventions. Trials should examine the timing and sequencing of surgery, medical optimisation, rehabilitation, lifestyle intervention, geriatric input, and digital follow-up. Future reviews should use prospectively documented search and screening procedures, formal risk-of-bias assessment, and certainty-of-evidence grading where appropriate. Standardised outcomes and pragmatic multicentre designs would improve comparability and support stronger guideline development.

## Conclusions

This narrative review indicates that contemporary general medicine and trauma emergency care require a multidisciplinary approach that extends beyond immediate survival to include complication prevention, functional recovery, chronic disease control, secondary prevention, and continuity of care. Evidence from trauma triage, resuscitation, emergency surgery, perioperative medicine, critical care, pain management, rehabilitation, preventive care, and digital follow-up supports the view that patient outcomes are influenced by coordinated decision-making across specialties rather than by isolated interventions. Surgical treatment remains essential for selected acute conditions, but its effectiveness depends on concurrent management of frailty, comorbidities, infection, organ dysfunction, medication exposure, and postoperative complications. Rehabilitation and lifestyle-based interventions may support functional recovery and long-term risk reduction, although their timing, intensity, and applicability should be individualised.

Several interventions reviewed produced mixed or neutral findings, including prehospital tranexamic acid, prehospital blood-product resuscitation, REBOA, restrictive fluid strategies, sedative selection, and early active mobilisation. These findings underscore the importance of careful patient selection, physiology-guided management, and avoidance of rigid, one-size-fits-all protocols. Integrated care should therefore be adapted to the patient's clinical condition, physiological reserve, comorbidity burden, functional goals, and available healthcare resources. Overall, coordinated care involving emergency medicine, surgery, internal medicine, critical care, rehabilitation, geriatrics, digital health, and preventive medicine may improve patient outcomes when implemented through individualised, evidence-informed pathways.

## Figures and Tables

**Figure 3:**
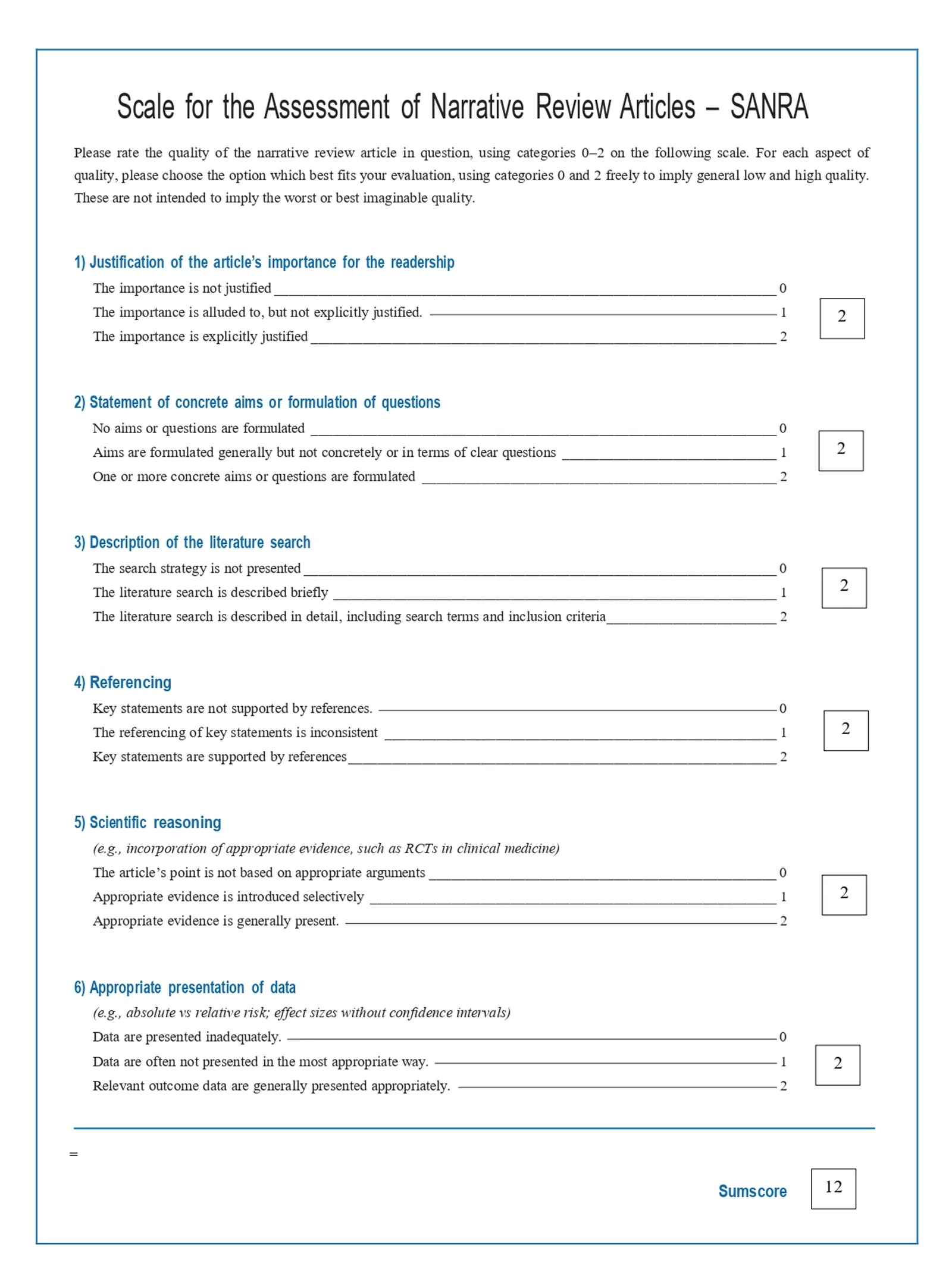
Scale for the Assessment of Narrative Review Articles checklist
